# Digital Assessment of Metacognition Across the Psychosis Continuum: Measures, Validity, and Clinical Integration—A Scoping Review

**DOI:** 10.3390/medicina62040704

**Published:** 2026-04-07

**Authors:** Vassilis Martiadis, Fabiola Raffone, Salvatore Clemente, Antonietta Massa, Domenico De Berardis

**Affiliations:** 1 Department of Mental Health, Asl Napoli 1 Centro, 80125 Naples, Italy; 2 Department of Psychiatry, University of Campania Luigi Vanvitelli, 80134 Naples, Italy; 3 Department of Mental Health, Asl Teramo, 64100 Teramo, Italy

**Keywords:** psychosis, schizophrenia, metacognition, cognitive insight, introspective accuracy, confidence, ecological momentary assessment, experience sampling, digital phenotyping, passive sensing

## Abstract

*Background and Objectives:* Metacognition-related processes (e.g., confidence calibration, self-evaluation and the use of feedback) have been linked to cognitive insight, self-evaluation, and daily functioning in psychosis. However, clinic-based assessments only provide limited information. Digital methods may capture state-like variations and contextual factors, but it is unclear to what extent they operationalise core metacognitive monitoring constructs versus adjacent self-evaluative/insight-related constructs. We mapped digital approaches used to assess metacognition-related constructs across the psychosis spectrum, summarising the associated feasibility and validity. *Materials and Methods:* We conducted a scoping review (PRISMA-ScR) of psychosis-spectrum studies that used digital tools to assess metacognition-related targets. These included ecological momentary assessment/experience sampling (EMA/ESM), task-based paradigms with confidence ratings, and hybrid approaches. Searches covered MEDLINE (via PubMed), Scopus, and IEEE Xplore, with the final search run on 15 December 2025. We charted constructs, operationalisations, feasibility/engagement indices and reported links with clinical or functional measures. *Results:* The empirical evidence map comprised 13 studies directly assessing metacognition-related constructs; eight additional implementation/methodological sources were synthesised separately to contextualise feasibility, reporting, ethics, and governance. EMA studies more often assessed adjacent self-evaluative constructs, including context-linked self-appraisal bias, conviction, and self-report–context mismatch in daily life, whereas task-based studies more directly assessed confidence–accuracy calibration and feedback updating. Across EMA studies, greater momentary symptom severity and more restricted contexts were often associated with inflated self-evaluations and divergence from observer-rated functioning. Task-based studies indicated that confidence calibration and feedback utilisation may diverge from objective performance; in performance-controlled paradigms, some studies reported comparable metacognitive sensitivity/efficiency, but the overall evidence remains uncertain. Passive sensing was common in psychosis research but was rarely explicitly tied to metacognitive constructs. *Conclusions:* Current digital work spans both core metacognitive monitoring constructs and adjacent self-evaluative/insight-related constructs, rather than a single unitary construct. Clinical translation remains hypothesis-generating: interpretability may be improved by combining clinical anchors, low-burden EMA, and optional contextual streams, but thresholds, workflows, and signal-action rules require prospective validation.

## 1. Introduction

Psychosis-spectrum disorders are clinically heterogeneous conditions with variable onset, trajectories and functional outcomes. Assessment strategies are required that capture both trait-like vulnerabilities and clinically meaningful fluctuations in daily life [1]. Metacognition, broadly defined as the capacity to monitor and evaluate one’s own mental processes and regulate behaviour based on such monitoring, has been linked to symptom expression, functional outcomes and recovery-oriented endpoints in schizophrenia spectrum disorders [2,3,4]. However, metacognition is not a single construct; rather, it comprises cognitive insight (self-reflectiveness and self-certainty), metacognitive beliefs and task-based metacognitive monitoring (e.g., confidence calibration), each with distinct conceptual and psychometric assumptions [5,6,7]. In this review, ‘metacognition-related’ is used as an umbrella term. Within that umbrella, we distinguish core metacognitive monitoring constructs (e.g., confidence–accuracy calibration and feedback updating) from adjacent self-evaluative or insight-related constructs (e.g., cognitive insight, self-appraisal bias, conviction/distress, and self-report–context mismatch), each with different conceptual and psychometric assumptions. Most conventional assessments of metacognition-related constructs are administered at discrete clinic visits and rely on retrospective reports or decontextualised performance. This creates a mismatch with the clinical reality of psychosis, where self-appraisal, conviction, affect, and social context can shift over hours to days and where functional decision-making often depends on momentary calibration (e.g., knowing when to seek help, interpreting anomalous experiences, and trusting internal judgements). Metacognitive interventions have been developed to modify reasoning biases, enhance insight, and improve reflective capacity. Meta-analytic evidence suggests that metacognitive training can improve certain aspects of cognitive insight, particularly self-reflectiveness, although the effects vary across trials [8]. More broadly, treatments targeting insight in psychosis have historically produced modest effects, motivating the search for mechanism-focused approaches [9].

Digital methods provide tools that can help to bridge this gap. Experience sampling methodology (ESM) and ecological momentary assessment (EMA) allow for repeated measurements in natural settings, which reduces recall bias and supports the analysis of the temporal relationship between affect, context and symptoms [10]. In psychosis research, EMA has been applied across diverse protocols. A systematic review and meta-analysis reported generally high completion rates while also documenting substantial variability in sampling designs and reporting practices [11]. Beyond active self-reporting, digital phenotyping uses data from smartphones and connected devices to quantify behaviour in situ and has expanded rapidly in psychosis-spectrum research for remote monitoring and the identification of markers relevant to relapse [12,13]. However, ethical and governance concerns remain central, and consensus work in mental health digital phenotyping emphasises privacy, transparency, consent, accountability, and bias as core requirements [14].

Despite the parallel growth of metacognition research and digital psychiatry, the extent to which these fields overlap remains unclear. Digital studies in psychosis often focus on symptom tracking, mobility, sleep or service use, whereas metacognition syntheses typically describe clinic-based scales or laboratory paradigms. Consequently, it is unclear which digital approaches genuinely assess either core metacognitive monitoring constructs or adjacent self-evaluative/insight-related constructs, and how these targets are operationalised (e.g., confidence calibration, feedback updating, cognitive insight, conviction, or self-report–context mismatch). There is also a lack of evidence regarding the feasibility, validity and clinical interpretability of these approaches. This scoping review maps digital operationalisations across this boundary and outlines cautious implementation considerations for digital assessment in psychosis, rather than proposing a validated clinical framework.

## 2. Materials and Methods

### 2.1. Design and Reporting

**Figure 1:**
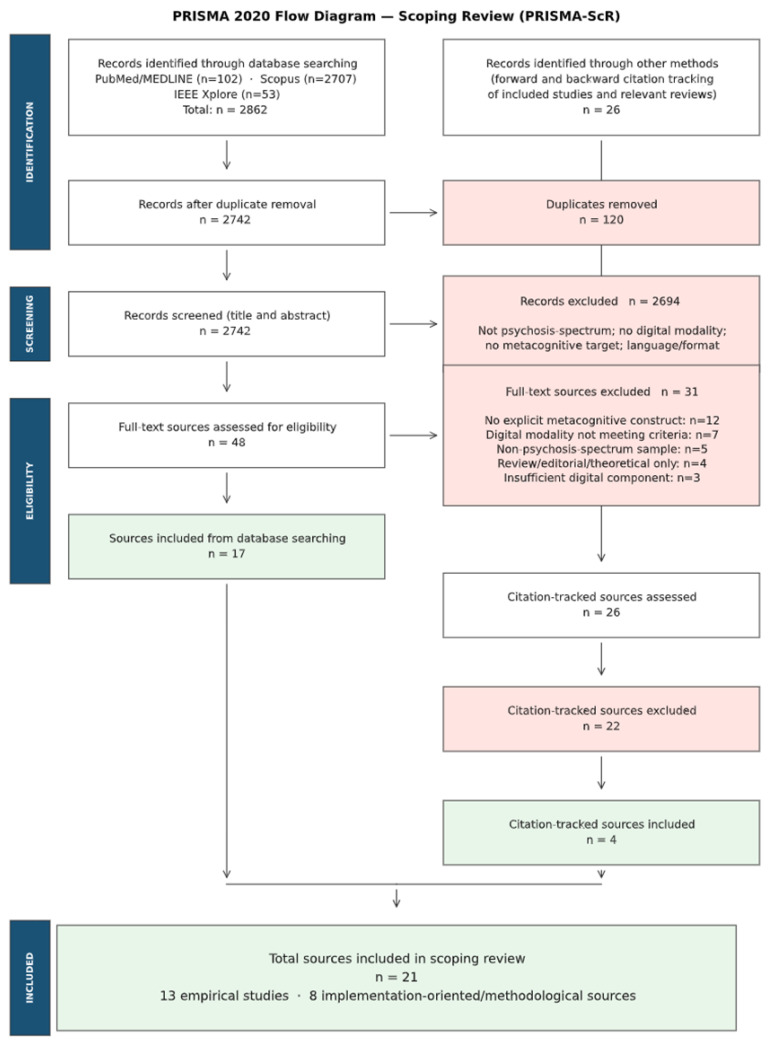
PRISMA 2020 flow diagram.

This scoping review aimed to map the extent, characteristics and gaps in the empirical evidence on the digital assessment of metacognition-related constructs in psychosis-spectrum populations. Reporting adhered to the PRISMA-ScR guidelines [15] and methodological guidance for scoping reviews [16]. In line with the journal’s reporting requirements for evidence syntheses, the PRISMA 2020 flow diagram is presented in Figure 1 of the main manuscript, and the completed PRISMA 2020 checklist is provided in the Supplementary Materials (Table S1). The protocol was not registered on external platforms.

### 2.2. Eligibility Criteria

Eligibility was defined using the Population–Concept–Context framework. Population: studies with participants diagnosed with schizophrenia, schizoaffective disorder, other primary psychotic disorders, first-episode psychosis or clinical high-risk/ultra-high-risk states. Mixed samples were included if psychosis-spectrum data were reported separately. Concept: studies were eligible if they assessed either (a) core metacognitive monitoring constructs, defined as trial-level monitoring of performance through confidence–accuracy calibration/metacognitive sensitivity or confidence updating after feedback; or (b) adjacent self-evaluative constructs explicitly framed by study authors as metacognition-, insight-, or self-monitoring-related, including cognitive insight, self-appraisal bias, delusional conviction/distress, affective self-awareness, and self-report–context mismatch. Studies assessing symptoms, mood, activity, service use, or passive-sensing features without an explicit metacognition, insight, or self-evaluative target were excluded. In the synthesis, core and adjacent constructs were reported separately, while passive/contextual streams were treated as contextual or engagement indicators rather than direct metacognitive measures. Context: digital assessment modalities included (i) active EMA/ESM; (ii) digital/computerised tasks incorporating confidence/accuracy judgements or feedback-based metacognitive indices; and (iii) passive or hybrid approaches, either when passive streams were used to contextualise metacognitive self-appraisal, or when metacognition-related targets were explicitly stated. Quantitative, qualitative and mixed-methods empirical studies were included, while editorials and purely theoretical papers were excluded. Review articles were not included in the evidence map but were used for citation chasing. Only English-language publications were considered.

### 2.3. Information Sources and Search Strategy

Searches were performed in the following databases: MEDLINE (via PubMed), Scopus, and IEEE Xplore. Forward and backward citation tracking of the included studies and relevant reviews was also conducted.

The search combined free-text terms and, where supported, controlled vocabulary to identify studies relating to (i) psychosis-spectrum populations, (ii) metacognition-related targets and (iii) digital assessment modalities (e.g., EMA/ESM, smartphone, wearable and passive sensing technologies, and digital phenotyping). The search strategy was adapted for each database using its syntax and field tags. The core database-specific strategies are reported below, and the complete strategies (including controlled vocabulary where applicable) are provided in Supplementary File S1. Search filters were limited to the English language and human studies where supported by the database interface.

PubMed (MEDLINE): (psychosis OR schizophrenia OR schizoaffective OR “first episode psychosis” OR “clinical high risk” OR UHR OR CHR) AND (metacogn* OR “cognitive insight” OR self-reflect* OR confidence OR calibration OR “error awareness” OR “introspective accuracy” OR “feedback updating”) AND (“ecological momentary assessment” OR EMA OR “experience sampling” OR ESM OR smartphone OR mobile OR app OR wearable OR “digital phenotyping” OR “passive sensing” OR computerized).

Scopus: TITLE-ABS-KEY ((psychosis OR schizophrenia OR schizoaffective OR “first episode psychosis” OR “clinical high risk”) AND (metacogn* OR “cognitive insight” OR confidence OR calibration OR “introspective accuracy” OR “feedback updating”) AND (“ecological momentary assessment” OR EMA OR “experience sampling” OR ESM OR smartphone OR app OR wearable OR “digital phenotyping” OR “passive sensing”)).

IEEE Xplore: (psychosis OR schizophrenia) AND (metacogn* OR “cognitive insight” OR confidence OR calibration OR “introspective accuracy” OR “feedback updating”) AND (“ecological momentary assessment” OR EMA OR “experience sampling” OR smartphone OR “digital phenotyping” OR “passive sensing” OR wearable). The final search date was 15 December 2025.

### 2.4. Study Selection

The records were de-duplicated prior to screening. Two reviewers conducted title/abstract and full-text screening independently, resolving disagreements through discussion. Rayyan was used to manage the screening process and any conflicts that arose [17].

### 2.5. Data Charting and Data Items

A standardised data-charting form was used and refined iteratively. The following items were extracted: publication year and country; design and setting; sample characteristics (e.g., diagnostic group, illness stage and age); digital modality (e.g., EMA/ESM, task-based or passive/hybrid); sampling schedule and duration; metacognitive construct and operationalisation; feasibility and acceptability metrics (e.g., completion rate, attrition rate and burden); validity evidence (e.g., convergent/discriminant associations and associations with clinical or functional outcomes); and reporting of ethical and governance elements (e.g., consent, privacy/security and data access).

### 2.6. Reporting Completeness Annotation

Consistent with scoping review methodology, we did not perform a formal risk-of-bias assessment of included studies. Studies were not excluded based on their methodological quality. To inform interpretation, the completeness of reporting was summarised descriptively using established digital health reporting guidance where applicable, including the mERA checklist [18] and relevant items from the Consolidated Standards of Reporting Trials (CONSORT) extension for e-Health (CONSORT-EHEALTH) [19].

### 2.7. Synthesis Approach

The findings were synthesised descriptively and organised according to digital modality, targeted construct and clinical interpretability. No quantitative pooling was performed due to the heterogeneity of the constructs, tools, and designs. To maintain the explicit boundaries of the corpus, we employed a two-part synthesis. First, we mapped empirical studies that directly assessed metacognition-related constructs in psychosis-spectrum samples. Second, we summarised contextual implementation and methodological sources separately, addressing issues of feasibility, adherence, privacy, data quality, ethics and reporting standards that are relevant to the interpretation or future deployment of digital assessment. As these contextual sources do not contribute to construct-level claims about metacognitive findings, they are presented separately from the empirical evidence base.

## 3. Results

Across the included studies, digital approaches mainly captured metacognition-related phenomena in two ways: (i) real-world self-appraisal, which was measured repeatedly using ecological momentary assessment/experience sampling (EMA/ESM); and (ii) confidence-based judgements during digital tasks, including changes in confidence after receiving performance feedback. Although passive sensing and broader digital phenotyping were common in psychosis research, they were rarely used to explicitly measure metacognition; in the eligible literature, passive streams mainly provided contextual information (e.g., sleep, mobility and time spent at home) or engagement indicators [12,13].

### 3.1. EMA/ESM: Adjacent Self-Appraisal and Conviction-Related Constructs in Daily Life

The included corpus of EMA/ESM studies predominantly assessed adjacent self-evaluative constructs rather than core metacognitive monitoring. These included self-appraisal of competence, affective self-awareness, delusional conviction/distress and self-report–context mismatch. While such constructs are clinically relevant, they should not be considered psychometrically interchangeable with confidence-based calibration measures.

Studies using EMA/ESM sampled symptoms, mood and self-evaluations multiple times per day. This allowed self-appraisal to be interpreted alongside daily context (e.g., being at home or alone). A recurring pattern across studies was that self-evaluations appeared to be weakly anchored to context when symptoms were more salient.

For instance, higher momentary psychotic symptom severity and more restricted contexts (e.g., home/alone) were linked to higher self-rated competence in everyday areas, suggesting a lack of sensitivity to context during periods of greater symptom burden [20]. Studies comparing self-ratings with observer ratings or behaviour-derived indicators have shown that observer ratings tend to track EMA-derived behaviour more closely than patient self-reports. Furthermore, very low reported sadness has been found to be paired with inflated self-evaluations [21,22]. Previous EMA research has also demonstrated that daily-life sampling can capture performance evaluations during social interactions and the dimensional aspects of delusions (including conviction and distress). This supports the feasibility of measuring appraisal-like constructs in naturalistic settings [23,24].

### 3.2. Task-Based Paradigms: Core Metacognitive Monitoring Constructs

Task-based paradigms usually involve participants making a decision and then rating their confidence (or judging whether they are correct), sometimes before being shown the correct answer. These designs quantify metacognitive monitoring at the trial level (i.e., how well confidence correlates with accuracy) and test feedback updating (i.e., whether confidence adjusts when informed of a response’s accuracy). In a meta-cognitive Wisconsin Card Sorting Test (WCST) variant, people with schizophrenia had less accurate confidence ratings and appeared to rely less on external feedback when updating judgements than people with bipolar disorder or healthy controls [25]. Similar patterns were reported in studies using trial-by-trial modelling of confidence, accuracy and feedback. These studies revealed attenuated feedback-related adjustments in schizophrenia in certain task conditions [26,27].

### 3.3. When Performance Is Controlled: Apparent Deficits May Be Attenuated, but Evidence Remains Uncertain

Not all task studies indicated a metacognitive deficit. In perceptual and memory paradigms that controlled for first-order performance, comparable levels of metacognitive sensitivity/efficiency were reported between schizophrenia spectrum samples and controls [28,29]. This finding is consistent with meta-analytic evidence showing that conclusions about metacognition in schizophrenia depend on how first-order performance differences are handled and modelled [3].

### 3.4. Feasibility and Reporting

Evidence of feasibility was most robust for EMA in psychosis. A recent systematic review and meta-analysis reported generally acceptable completion rates but also highlighted variations in sampling intensity and limited reporting of item validation and missing data handling procedures [11]. For passive, smartphone-based approaches, acceptability may present a challenge: one study found that uptake was low, but retention among those who engaged was higher. This highlights the importance of trust, privacy, and perceived benefit [30]. Across the broader passive sensing literature, inconsistent preprocessing and reporting practices remain common, which limits comparability and slows translation to routine settings [13].

**Figure 2:**
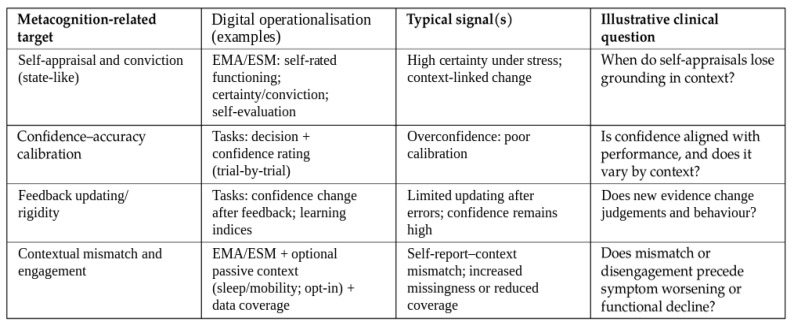
Pragmatic map of metacognition-related targets and digital operationalisations identified in psychosis-spectrum studies. The map is descriptive and reflects how included studies framed and measured targets; it does not imply established clinical validity. Bracketed numbers indicate exemplar references.

Overall, the available evidence suggests that current digital assessments mainly capture adjacent self-evaluative constructs in EMA and core metacognitive monitoring constructs in task-based paradigms, with purely passive proxies of metacognitive processes being uncommon in the included studies. Key empirical studies are summarised in Table 1, while complementary implementation-oriented sources are summarised in Table 2. A pragmatic construct map is shown in Figure 2.

## 4. Discussion

Digital methods are increasingly being used to study metacognition-related processes in psychosis outside of clinical settings. The two most common approaches in the evidence mapped here are (i) repeated self-ratings in daily life (EMA/ESM), which often capture adjacent self-evaluative constructs, including self-appraisal bias and self-report–context mismatch, and (ii) task-based paradigms that quantify confidence and the use of feedback during performance. A recurring issue is construct drift: some protocols use broad labels such as ‘insight’ or ‘metacognition’ when measuring only symptoms or activity. When digital measures specify the target more clearly (e.g., confidence calibration, self-report–context mismatch, or feedback updating), the findings are easier to interpret and more likely to inform clinical decisions. In routine care, it is often more important to understand whether self-evaluations remain grounded when stress or symptoms increase than to obtain a single trait score.

### 4.1. ESM: Self-Appraisal and Self-Report–Context Mismatch in Context

Early studies reported that computerised EMA could be feasible in schizophrenia and could capture phenomena relating to daily life that are also reflected in laboratory measures [34]. More recent syntheses in psychosis support the feasibility of EMA at the group level, with generally acceptable completion rates. However, they also point to wide differences in sampling schedules, study duration and the psychometric validation of many EMA items [11]. These features limit comparability across studies and make it difficult to define a ‘good enough’ measurement for clinical use.

A clinically relevant pattern emerging from EMA studies is that subjective self-evaluations can become less aligned with context and objective constraints. For instance, higher momentary psychotic experiences have been linked to a tendency to overestimate competence in everyday areas, suggesting that worsening symptoms may coincide with reduced plausibility checking in self-appraisal [20]. Related work has shown that some participants report persistently low negative affect while simultaneously endorsing inflated functioning and spending more time at home alone. This combination may appear reassuring in a brief interview, but it may also indicate reduced access to internal states and social withdrawal in daily life [21]. These findings suggest that EMA could help clinicians to move away from making a global judgement and towards providing a more specific description of self-appraisal becoming less grounded in certain situations. In practice, discussing concrete patterns (e.g., ‘high confidence on days with high stress and low activity’) may facilitate a shared understanding, reduce blame and guide collaborative goal setting, particularly when data are presented as trends rather than verdicts [20,21,22]. Nevertheless, before interpreting self-report–context mismatch as reduced metacognitive grounding, several alternative explanations need to be considered. Divergence between self-rated competence and contextual/observer indicators may reflect negative symptoms, a depressive reporting style, neurocognitive impairment, a lack of opportunity or environmental deprivation, different personal values regarding the activity in question, or observer/informant error. For this reason, EMA-derived mismatch should be interpreted as a signal that requires further investigation rather than as a standalone indicator.

### 4.2. Task-Based Digital Paradigms: Confidence Dynamics and Feedback Updating

Task-based paradigms provide complementary information by distinguishing between first-order performance (providing the correct answer) and second-order monitoring (evaluating confidence levels and changes in confidence following feedback). Studies using confidence ratings, metacognitive efficiency indices or feedback-based learning can operationalise monitoring and updating in a manner closer to formal metacognitive models [25,26,27]. However, tasks differ markedly in terms of difficulty, feedback structure and analytic choices, which limits direct comparison.

Time-series and trial-level analyses further suggest that impairment may not always be random or ‘noisy’. Instead, some patterns resemble rigidity, such as confidence remaining high despite errors or feedback not sufficiently adjusting future judgements. These dynamics may be clinically relevant because they can be mapped onto how certain beliefs persist in the face of contradictory experiences. Intensive designs, including experience sampling within trials, may detect changes in pathways (e.g., the relationship between emotion and paranoia) even when overall symptom changes are modest [31].

A key interpretive caveat concerns first-order performance. A 2021 meta-analysis identified an overall metacognitive deficit in schizophrenia but also indicated that the magnitude of this deficit was exaggerated in studies that did not equate first-order performance [3]. Therefore, task-based readouts should be interpreted alongside first-order performance, task demands, reward structure, fatigue and analytical choices. Similarly, updating confidence on a task in a limited way may reflect poorer initial learning, the difficulty of the task, fatigue, sensitivity to reward, or broader executive dysfunction, rather than a distinct mechanism of metacognitive rigidity.

### 4.3. Passive Sensing and Digital Phenotyping: Contextual Signals, Data Quality, and Governance

Although passive sensing and digital phenotyping have expanded rapidly in psychosis research, explicit linkage to core metacognitive monitoring or adjacent self-evaluative constructs remains uncommon. Recent systematic reviews demonstrate the existence of extensive and growing bodies of literature on passive data streams (e.g., activity, sleep and mobility) and machine learning pipelines in psychosis. However, there is substantial heterogeneity in devices, preprocessing, feature definitions and reporting [10,12,13]. Consequently, passive signals are better understood as contextual information (i.e., what is happening in daily life) than as direct measures of metacognition.

From an implementation perspective, two issues are central. Firstly, passive data only become clinically usable when the path from raw data to derived features to inferences is transparent and missing data and data quality thresholds are reported in a replicable way across services [12,13]. Secondly, ‘data quality’ itself may carry information: reduced survey completion or sensor coverage may reflect disengagement, contextual barriers or worsening symptoms and can therefore be relevant to safety planning rather than being treated solely as technical noise [35].

Ethics and acceptability are equally important. People with psychosis often describe potential benefits, such as earlier support, monitoring sleep and reduced burden, as well as substantial concerns, such as surveillance, loss of control and stigma. Qualitative evidence emphasises the importance of choice regarding specific data streams and clear rules regarding data access and usage [37]. Similarly, broader consensus work in digital phenotyping highlights privacy, transparency, consent and accountability as non-negotiable requirements for responsible deployment [14].

### 4.4. Implementation Considerations and Evidentiary Limits

**Figure 3:**
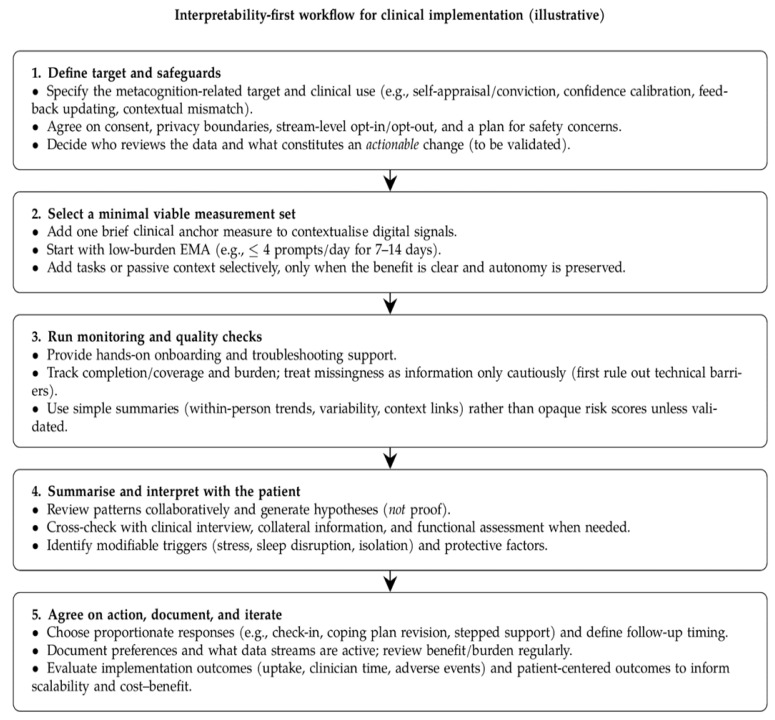
Interpreting digital monitoring findings in practice: an illustrative, author-derived framework for future testing. The figure is hypothesis-generating and does not represent a validated care pathway, decision rule, or evidence of clinical effectiveness. Passive streams, if used, should remain optional and patient-controlled.

Implementation studies suggest that adoption is influenced by trust, perceived benefit, burden, and practical support. Taken together, these findings indicate broad implementation considerations rather than a validated pathway. A low-burden sequence—starting with clearly explained active monitoring and adding optional passive streams only when patients perceive value and retain control—should therefore be understood as an illustrative option for future feasibility testing, not as a review-derived recommendation. Likewise, any response to digital signals or missingness requires local governance, prospective evaluation, and clinical judgement. Figure 3 summarises an author-derived interpretive framework intended to make these dependencies explicit rather than to prescribe care.

### 4.5. Illustrative Considerations for Future Testing

Digital assessment may become clinically useful when it produces simple, interpretable patterns that can be discussed with patients, rather than opaque composite scores. The points below are not recommendations supported by evidence of effectiveness. They are considerations derived by the authors and informed by feasibility studies, observational findings and early mechanistic work. They are intended to clarify what future implementation studies should test.

(1) Define the metacognitive target before choosing the tool. Clarify whether the question concerns cognitive insight, confidence calibration, self-report–context mismatch, or feedback updating, and match the digital measure accordingly to reduce construct drift [5,25,26,27].
(2) Use brief, supported EMA bursts when the aim is to capture change within individuals. While EMA is often feasible in psychosis, adherence can vary depending on the setting and the severity of the symptoms. Planning is required for onboarding, prompt burden and staff support, especially in residential settings [11,36].
(3) Consider self-report–context mismatch as a candidate interpretive signal rather than a standalone indicator. Recurrent divergence between self-rated competence and contextual/observer indicators may warrant collaborative review and functional assessment. However, it can also reflect negative symptoms, a depressive reporting style, neurocognitive impairment, environmental deprivation, differing personal values or a measurement artefact [20,21,22,24].
(4) Use task-based readouts to generate hypotheses tailored to the individual rather than treatment rules. When confidence remains high despite negative feedback or when updating appears limited, these patterns may warrant closer examination of doubt, flexibility, error awareness, first-order performance, task demands and measurement reliability. They should not be treated as diagnostic proof [25,26,27].
(5) Treat data quality as a technical and engagement variable. Missing EMA responses or reduced sensor coverage may indicate disengagement, contextual barriers or clinical deterioration and may necessitate a supportive review rather than automatic exclusion [35].
(6) Prefer autonomy-preserving governance. Data minimisation, stream-level opt-in/opt-out options, clear access rules and transparent communication about the intended use and limits of data may improve acceptability and reduce ethical risks [14,37].

One possible low-burden package for future feasibility testing could include the following: (i) a brief clinic-based anchor measure aligned with the selected target construct; (ii) a short EMA module (7–14 days, low burden) focused on self-appraisal and conviction; and (iii) a simple review template for summarising within-person patterns. This should be viewed as an illustrative option rather than a standard pathway. If used, passive streams should be added selectively, remain patient-controlled and be accompanied by a local feasibility evaluation.

Table 3 provides illustrative implementation considerations for future testing, and Table 4 provides illustrative collaborative review mappings.

The issues of scalability and cost–benefit remain open empirical questions. Although digital monitoring may seem unburdensome for patients, its routine use can necessitate a considerable amount of staff time for onboarding, troubleshooting, data review, governance and technical support. For this reason, any broader deployment would benefit from staged feasibility work to document the workload, dropout rate, adverse events, false alarms and net value before wider implementation.

### 4.6. Limitations

This review has limitations that are typical for scoping work. First core metacognitive monitoring constructs and adjacent self-evaluative/insight-related constructs were defined and measured in many different ways, and some studies relied on indirect proxies; this reduces comparability and is one reason why we did not pool results quantitatively. Second, studies were not excluded on the basis of formal risk-of-bias tools, and conclusions therefore depend on the design and reporting quality of the underlying evidence. Third, many digital studies reported limited validation against established metacognition measures, and passive sensing studies often provided insufficient detail on preprocessing and feature construction, which constrains interpretability. Fourth, database coverage was limited to MEDLINE (via PubMed), Scopus, and IEEE Xplore, as institutional access to Embase, PsycINFO, and the Web of Science Core Collection was unavailable. Studies indexed exclusively in those databases may have been missed; forward and backward citation tracking of all included sources was conducted to partially mitigate this gap. An additional limitation is that our boundary-mapping approach favoured sensitivity over conceptual specificity: some included EMA constructs were adjacent to, rather than direct measures of, core metacognitive monitoring. Finally, limiting inclusion to English-language publications may have missed relevant work.

## 5. Conclusions

Digital assessment in psychosis currently spans two partially overlapping domains: core metacognitive monitoring constructs, mainly assessed in task-based paradigms, and adjacent self-evaluative/insight-related constructs, more commonly assessed with EMA/ESM. This distinction is important for interpretation. Although the field shows promise, it remains at an early stage. Future work should prioritise construct specification, performance-controlled designs, transparent reporting, triangulation against clinical and cognitive anchors, and autonomy-preserving governance before digital signals are embedded in clinical workflows.

## Figures and Tables

**Table 1:** Key empirical studies examining digital assessment of metacognition-related constructs in psychosis-spectrum samples. (Ordered chronologically by year of publication).

Study	Sample	Digital Modality	Metacognition Target	Main Finding
Ben-Zeev et al., 2012 [24]	Schizophrenia/schizoaffective (community-dwelling, N = 130)	PDA-based EMA (multiple prompts/day for 7 days)	Delusional dimensions (occurrence, conviction, distress, disruption)	Delusions were decomposed into distinct momentary dimensions; predictors differed by subtype, with hallucinations predicting subsequent delusional experiences and negative self-esteem relating to delusion frequency [24].
Granholm et al., 2013 [23]	Schizophrenia (outpatients)	EMA/ESM (daily-life sampling)	Performance appraisals in social interactions	Momentary appraisals and affect were linked to subsequent social interaction patterns, supporting ecological assessment of self-evaluations relevant to functioning [23].
Pos et al., 2018 [31]	Recent-onset psychosis (RCT, N = 50)	EMA/ESM + questionnaires	Cognitive insight; affect–paranoia coupling	No mean-level superiority of MCT vs. active control on paranoia/cognitive insight; altered affect–paranoia coupling post-treatment in MCT arm [31].
Durand et al., 2021 [21]	Schizophrenia (N = 102) vs. bipolar (N = 71)	EMA (3/day, 30 days)	Self-report bias vs. behaviour	Observer ratings tracked EMA-derived behaviour; self-reports diverged. Very low sadness reports linked to inflated self-reported functioning [21].
Faivre et al., 2021 [28]	Schizophrenia/schizoaffective (N = 20) vs. controls (N = 21)	Computerised perceptual task	Metacognitive sensitivity (confidence–accuracy)	In this small performance-controlled perceptual paradigm, Bayesian analyses did not detect a clear metacognitive deficit [28].
Jones et al., 2021 [22]	Schizophrenia vs. bipolar	EMA (up to 90 surveys)	Affective self-awareness; self-assessment bias	Subgroup reporting “never sad” showed inflated self-assessed functioning and more home/alone time, without parallel objective deficits [22].
Tercero et al., 2021 [25]	Schizophrenia (N = 99) vs. bipolar (N = 67)	Digital task (meta-WCST)	Introspective accuracy; confidence; feedback use	In schizophrenia, confidence tracked self-generated judgments more than objective performance; weaker incorporation of feedback [25].
Gohari et al., 2022 [20]	Schizophrenia (N = 101)	EMA (3/day, 30 days)	Self-assessment of competence; plausibility checking	Higher momentary psychotic symptoms and being home/alone associated with inflated self-rated competence, suggesting context-insensitive self-appraisal [20].
Badal et al., 2023a [26]	Schizophrenia (N = 144) vs. bipolar (N = 140) vs. controls (N = 39)	Digital tasks + time-series networks	Confidence–accuracy–feedback dynamics	Weaker influence of feedback on subsequent confidence/accuracy in schizophrenia; higher confidence predicted worse subsequent WCST performance in schizophrenia [26].
Badal et al., 2023b [27]	Schizophrenia spectrum	Digital tasks (seasonal decomposition)	Dynamics of confidence over trials	Reduced adaptive variability in task-based confidence, suggesting impairments in flexible monitoring [27].
Rouy et al., 2023a [29]	Schizophrenia vs. controls	Computerised tasks (perception/memory) + confidence	Metacognitive sensitivity/efficiency controlling performance	No clear specific metacognitive deficit was detected across tasks under performance-matched conditions [29].
Rouy et al., 2023b [32]	Schizophrenia spectrum	EEG + confidence task	Neural markers of confidence/performance monitoring	No clear alteration in electrophysiological confidence markers under performance matching; underscores first-order performance confounding [32].
Gorora et al., 2024 [33]	Schizophrenia (N = 99) vs. bipolar (N = 76)	Digital task (meta-WCST)	Momentary accuracy decisions pre-feedback	Lower correct accuracy decisions and positive response bias; results consistent with reduced use of external feedback in schizophrenia [33].

**Table 2:** Contextual implementation and methodological sources used to interpret feasibility, ethics, governance, and reporting (not part of the empirical evidence map).

Source	Focus	Key Message	Clinical Relevance
Granholm et al., 2008 [34]	Computerised EMA feasibility/validity in schizophrenia	High compliance over one week and correlations with laboratory measures [34].	Supports feasibility of intensive sampling when burden and support are adequate.
Torous et al., 2018 [35]	Data quality in digital phenotyping in schizophrenia	Sensor coverage and completion metrics associated with subsequent symptom survey scores [35].	Missingness can be clinically meaningful and should trigger review.
Lopez-Morinigo et al., 2021 [30]	Acceptability of passive smartphone-based EMA app (eB2)	Initial uptake was limited; retention among adopters was higher [30].	Adoption depends on trust, perceived benefit, and onboarding rather than “passivity” alone.
Martinez-Martin et al., 2021 [14]	Ethics consensus for digital phenotyping	Privacy, transparency, consent, accountability, and bias prioritised [14].	Metacognition monitoring must be negotiated to avoid surveillance harms.
Zarbo et al., 2023 [36]	ESM adherence patterns and predictors (DiAPAson)	Adherence differed by setting and was lower with more severe positive symptoms; collaboration skills predicted better adherence [36].	Implementation requires tailoring protocols and providing support, especially in residential services.
Bell et al., 2024 [11]	EMA feasibility in psychosis (systematic review/meta-analysis)	Completion rates generally high but protocols and reporting variable [11].	Supports feasibility; underscores tailoring burden and standardising reporting.
Bladon et al., 2025 [13]	Passive data processing in psychosis (systematic review)	Marked heterogeneity in passive streams, preprocessing, and features [13].	Passive context can inform mismatch only if pipelines are transparent.
Eisner et al., 2025 [37]	Qualitative views on passive sensing for relapse prediction	Benefits valued but autonomy/privacy concerns prominent; preference for choice/control [37].	Clinical deployment should prioritise opt-in streams and understandable feedback.

**Table 3:** Author-derived implementation considerations for future testing in psychosis services (illustrative only; not review-derived recommendations; requires local validation).

Implementation Domain	Illustrative Consideration(s) for Future Testing
Clinical aim and target construct	Specify the metacognition-related target (e.g., cognitive insight, self-appraisal/conviction, confidence–accuracy calibration, feedback updating, self-report–context mismatch) and the clinical decision it is meant to inform.
Minimal viable measurement set	Use a brief clinical anchor (one validated insight/metacognition measure) to keep digital signals interpretable; start with low-burden EMA (≤4 prompts/day for 7–14 days) before adding tasks or passive streams.
Workflow and interpretability	Predefine who reviews the data, how often, and what triggers a clinical contact. Prefer simple summaries (trends, within-person changes) over opaque risk scores unless validated.
Participant-centred onboarding and burden management	Provide hands-on onboarding, written instructions, and a contact for troubleshooting. Negotiate notification schedules; allow pauses; monitor burden and drop-out early.
Governance, privacy, and autonomy	Use data minimisation and stream-level opt-in/opt-out (especially for location). Clarify data access, storage, and limits of confidentiality; avoid ‘surveillance’ framing.
Equity and accessibility	Assess digital literacy and device access; offer alternatives (paper, phone calls) or device loan where feasible. Ensure language accessibility and accommodations for cognitive difficulties.
Scalability and cost–benefit	Plan for staff time (onboarding, review), IT support, and maintenance. Collect implementation outcomes (uptake, adherence, clinician time) and patient-centred outcomes to inform cost–benefit decisions.

**Table 4:** Candidate digital signals and possible collaborative review responses for future testing (illustrative only; not evidence of effectiveness or a validated workflow).

Potential Digital Signal (Candidate)	Possible Collaborative Review Response (Illustrative; To Be Validated)
Abrupt increase in conviction/distress ratings or rapid within-day volatility in belief certainty (EMA)	Collaborative review of context, stressors, and safety; revisit coping plan; consider whether closer review or support is warranted according to existing service procedures.
Persistent high confidence despite repeated task errors (poor calibration) in brief digital tasks	Use as a starting point for metacognitive dialogue; review whether first-order performance, task difficulty, fatigue, or misunderstanding could explain the pattern before drawing inferences.
Limited confidence updating after corrective feedback (rigidity) during task performance	Explore how feedback is understood; consider whether the pattern reflects learning demands, fatigue, reward sensitivity, or executive dysfunction as well as possible metacognitive inflexibility.
Self-report–context mismatch (e.g., high self-rated functioning but marked isolation/low activity) when passive context is opt-in	Review the discrepancy with the patient; conduct a functional assessment; consider alternative explanations including negative symptoms, depressive reporting style, contextual deprivation, reduced grounding of self-appraisal stigma, and measurement artifacts.
Progressive increase in missing EMA prompts or drop in sensor coverage (data quality/engagement)	First, assess technical barriers and burden; if feasible, treat sustained missingness as a ‘signal’ warranting a supportive contact and risk review, rather than imputing it away.
Patient opts out of a stream or reports privacy concerns	Respect the choice; offer lower-intrusion alternatives and re-negotiate goals. Document preferences and ensure transparency about what is and is not being collected.

## Data Availability

No new datasets were generated for this review. Data sharing is not applicable.

## Supplementary Materials

The following supporting information can be downloaded at: https://www.mdpi.com/article/10.3390/medicina62040704/s1, Supplementary File S1: Full database-specific search strategies; Table S1: PRISMA Extension for Scoping Reviews (PRISMA-ScR) Checklist.

## Abbreviations

The following abbreviations are used in this manuscript:

BCIS	Beck Cognitive Insight Scale
CHR	Clinical high risk
EMA	Ecological momentary assessment
ESM	Experience sampling methodology
FEP	First-episode psychosis
mERA	mHealth Evidence Reporting and Assessment
MCT	Metacognitive training
PDA	Personal digital assistant
PRISMA-ScR	PRISMA Extension for Scoping Reviews
UHR	Ultra-high risk
