# Supplementary material for: Digital Assessment of Metacognition Across the Psychosis Continuum: Measures, Validity, and Clinical Integration—A Scoping Review
Source: Medicina (Kaunas). 2026 Apr 7;62(4):704. doi: 10.3390/medicina62040704 (PMC13117360; doi:10.3390/medicina62040704)
Supplement: Supplementary file 1 [file medicina-62-00704-s001.zip › medicina-4225816-supplementary.pdf]

Supplementary File S1. Full database-specific search strategies

Searches were run from database inception to 15 December 2025 (final search date: 15 December 2025). The protocol was not registered. The search strategy was adapted to each database syntax and, when available, controlled vocabulary (MeSH/Emtree/APA Thesaurus terms). Limits: English language; Human studies (where available). The strings below reflect the strategies used for the scoping review.

PubMed (MEDLINE)

("Psychotic Disorders"[MeSH Terms] OR "Schizophrenia"[MeSH Terms] OR "Schizophrenia Spectrum and Other Psychotic Disorders"[MeSH Terms] OR psychosis[tiab] OR psychotic[tiab] OR schizophrenia[tiab] OR schizoaffective[tiab] OR "first episode psychosis"[tiab] OR "first-episode psychosis"[tiab] OR FEP[tiab] OR "clinical high risk"[tiab] OR "ultra high risk"[tiab] OR UHR[tiab] OR CHR[tiab]) AND ("Metacognition"[MeSH Terms] OR metacogn*[tiab] OR "cognitive insight"[tiab] OR self-reflect*[tiab] OR confidence[tiab] OR calibration[tiab] OR "error awareness"[tiab] OR "introspective accuracy"[tiab] OR "feedback updating"[tiab]) AND ("Ecological Momentary Assessment"[MeSH Terms] OR "Smartphone"[MeSH Terms] OR "Mobile Applications"[MeSH Terms] OR "Wearable Electronic Devices"[MeSH Terms] OR "experience sampling"[tiab] OR EMA[tiab] OR ESM[tiab] OR smartphone*[tiab] OR mobile[tiab] OR app*[tiab] OR wearable*[tiab] OR "digital phenotyping"[tiab] OR "passive sensing"[tiab] OR computerized[tiab])

Scopus

TITLE-ABS-KEY((psychosis OR schizophrenia OR schizoaffective OR "first episode psychosis" OR "clinical high risk") AND (metacogn* OR "cognitive insight" OR confidence OR calibration OR "introspective accuracy" OR "feedback updating") AND ("ecological momentary assessment" OR EMA OR "experience sampling" OR ESM OR smartphone OR app OR wearable OR "digital phenotyping" OR "passive sensing"))

IEEE Xplore

(psychosis OR schizophrenia) AND (metacogn* OR "cognitive insight" OR confidence OR calibration OR "introspective accuracy" OR "feedback updating") AND ("ecological momentary assessment" OR EMA OR "experience sampling" OR smartphone OR "digital phenotyping" OR "passive sensing" OR wearable)

Table S1. PRISMA Extension for Scoping Reviews (PRISMA-ScR) Checklist

Tricco AC et al. Ann Intern Med. 2018;169:467–473. https://doi.org/10.7326/M18-0850

Section	#	PRISMA-ScR item	Location in manuscript	Comment
TITLE	1	Identify the report as a scoping review.	Title	
ABSTRACT	2	Provide a structured summary including background, objectives, eligibility criteria, sources of evidence, charting methods, results, and conclusions.	Abstract	
INTRODUCTIO N	3	Describe the rationale for the review in the context of what is already known.	§1 Introduction	
	4	Provide an explicit statement of the objectives or questions the review addresses.	§1 Introduction (last paragraph)	
METHODS	5	Indicate whether a review protocol exists; state if and where it can be accessed; provide registration information.	§2.1	Protocol not registered.
	6	Specify characteristics of the sources of evidence used as eligibility criteria (PCC framework).	§2.2	
	7	Describe all information sources (e.g., databases, contact with authors) in the search and date last searched.	§2.3	
	8	Present the full electronic search strategy for at least one database, including any limits used.	§2.3 + Supplementary File S1	
	9	State the process for selecting sources of evidence.	§2.4	
	10	Describe the methods of charting data from the included sources of evidence.	§2.5	
	11	List and define all variables for which data were sought and any assumptions made.	§2.5	
	12	If done, describe methods for assessing methodological quality of included sources.	§2.6	Formal risk-of-bias not performed (scoping review methodology).

Section	#	PRISMA-ScR item	Location in manuscript	Comment
	13	Describe the methods of handling and summarising the data.	§2.7	
RESULTS	14	Give numbers of sources of evidence screened, assessed for eligibility, and included in the review, with reasons for exclusions at each stage, ideally using a flow diagram.	§3 + Figure S1	
	15	For each source of evidence, present characteristics for which data were charted and provide the citations.	Tables 1–2	
	16	If done, present data on critical appraisal of included sources of evidence.	§2.6	Reporting completeness annotated descriptively.
	17	For each included source of evidence, present the relevant data that were charted.	Tables 1–2; §3.1–3.4	
	18	Summarise and/or present the charting results as they relate to the review objectives.	§3; Figures 1–2	
DISCUSSION	19	Summarise the main results with reference to the review question.	§4 Discussion (opening)	
	20	Discuss the limitations of the scoping review process.	§4.6	
	21	Provide a general interpretation of the results with respect to other evidence, as well as implications for future research.	§4.1–4.5; §5 Conclusions	
FUNDING	22	Describe sources of funding for the included sources of evidence, as well as sources of funding for the scoping review. Describe the role of the funders.	Funding statement	No external funding.
